# PipeNig^®^-FL, a Fluid Extract of Black Pepper (*Piper Nigrum* L.) with a High Standardized Content of *Trans*-β-Caryophyllene, Reduces Lipid Accumulation in 3T3-L1 Preadipocytes and Improves Glucose Uptake in C2C12 Myotubes

**DOI:** 10.3390/nu11112788

**Published:** 2019-11-15

**Authors:** Federica Geddo, Rosaria Scandiffio, Susanna Antoniotti, Erika Cottone, Giulia Querio, Massimo E. Maffei, Patrizia Bovolin, Maria Pia Gallo

**Affiliations:** 1 Department of Life Sciences and Systems Biology, University of Turin, Via Accademia Albertina 13, 10123 Turin, Italy; federica.geddo@unito.it (F.G.); rosaria.scandiffio@unito.it (R.S.); susanna.antoniotti@unito.it (S.A.); erika.cottone@unito.it (E.C.); giulia.querio@unito.it (G.Q.); patrizia.bovolin@unito.it (P.B.); 2 Plant Physiology Unit, Department of Life Sciences and Systems Biology, University of Turin, Via Quarello 15/a, 10135 Turin, Italy; massimo.maffei@unito.it

**Keywords:** *trans*-β-caryophyllene, adipogenesis, lipid accumulation, glucose uptake, GLUT4, black pepper

## Abstract

*Trans*-β-caryophyllene (BCP) is a natural sesquiterpene hydrocarbon with several important pharmacological activities, including antioxidant, anti-inflammatory, anticancer, and cardioprotective functions. These properties are mainly due to its selective interaction with the peripherally expressed cannabinoid receptor 2. In addition, BCP activates peroxisome proliferated activator receptors α and γ and inhibits the Toll-like receptor signaling pathway. Given the growing scientific interest in BCP, the aim of our study was to investigate the metabolic effects of a black pepper extract (PipeNig^®^-FL), containing a high standardized content of BCP. In particular our interest was focused on its potential activity on lipid accumulation and glucose uptake. The extract PipeNig^®^-FL was chemically characterized by gas chromatography–mass spectrometry (GC–MS) and gas chromatography with flame-ionization detection (GC–FID), confirming a high content (814 mg/g) of BCP. Experiments were performed on 3T3-L1 preadipocytes and on C2C12 myotubes. Lipid content following 3T3-L1 adipogenic differentiation was quantified with AdipoRed fluorescence staining. Glucose uptake and GLUT4 membrane translocation were studied in C2C12 myotubes with the fluorescent glucose analog 2-NBDG and by immunofluorescence analysis. Here we show that PipeNig^®^-FL reduces 3T3-L1 adipocyte differentiation and lipid accumulation. Moreover, acute exposure of C2C12 myotubes to PipeNig^®^-FL improves glucose uptake activity and GLUT4 migration. Taken together, these results reveal interesting and novel properties of BCP, suggesting potential applications in the prevention of lipid accumulation and in the improvement of glucose uptake.

## 1. Introduction

Metabolic syndrome is a non-communicable disease characterized by visceral adiposity, insulin resistance, hyperlipidemia, hypertension and a chronic low-grade inflammatory state, often dragging into the onset of type 2 diabetes, coronary disease, stroke, and other disabilities. This chronic illness represents a major health hazard in the modern population, being rapidly spread from Western to developing countries [1]. The multiple biological mechanisms included in metabolic syndrome provide a complex interorgan communication, involving adipokines, macrophages, endoplasmic reticulum stress, thyroid hormones, beta adrenergic hormones, gut microbiome and other factors, where epigenetic drivers represent the major component with respect to genetic predisposition [1]. The pharmacological treatment of metabolic syndrome commonly involves anti-obesity drugs, thiazolidinediones (TZDs), metformin, statins, fibrates and several other drugs [2], but its management chiefly lies in the adoption of a healthy lifestyle [3]. In this perspective, many studies and clinical trials highlight some quality diets, such as the Mediterranean diet, the Nordic diet, and the Dietary Approaches to Stop Hypertension (DASH) diet, to protect against metabolic syndrome or to improve its phenotype. Moreover, plants and plant-derived molecules have received great attention as complementary supports in managing metabolic dysfunctions [4]. As an example, *Piper nigrum*, a widely used spice, has been present in traditional medicine of different countries all over the world since ancient times due to the beneficial effects of its biologically active extracts, underlining its possible use in the treatment of metabolic syndrome or other related conditions [5]. Among active compounds naturally present in *Piper nigrum*, the dietary cannabinoid *trans*-β-caryophyllene (BCP) could be considered as a possible key component in the treatment of obesity and type 2 diabetes in the metabolic syndrome scenario [6].

BCP is a bicyclic sesquiterpene hydrocarbon, highly present in a consistent number of plant-derived essential oils, such as balsams of *Copaiba* spp. (up to 53%), black pepper (*Piper nigrum*, up to 70%), lemon balm (*Melissa officinalis*, up to 19%), cloves (*Syzygium aromaticum*, up to 12%) and hops (*Humulus lupulus*, up to 9%) [7]. The Research Institute for Fragrance Materials (RIFM) evaluated BCP safety [8] and it has been approved by the Food and Drug Administration and by the European Food Safety Authority as a flavoring agent, usable in cosmetic and food additives. In recent years, many studies reported the beneficial properties of BCP against several disorders, in particular cancer, chronic pain and inflammation; among the main ones, recent findings showed chemosensitizing properties for doxorubicin chemotherapy [9], sorafenib [10], and 5-fluorouracil and oxaliplatin [11], neuroprotective effects against cerebral ischemia reperfusion injury [12] and dopaminergic neuron injury [13], cardioprotective features against myocardial infarction [14] and doxorubicin toxicity [15] and, especially, a significant impact in the metabolic syndrome context. BCP has indeed been highlighted as a hypocholesterolemic and insulinotropic agent in high-fat diet-fed [16,17], or in streptozotocin-induced, diabetic rats [18,19], where it showed non-clinical toxicity and an absence of adverse effects [20]. BCP can act as a selective agonist of cannabinoid receptor 2 (CB2) [21], can directly activate peroxisome proliferator-activated receptor-α (PPAR α) [22], involved mainly in liver metabolism, and triggers the activation of PPPAR γ [17], a master regulator of adipogenesis, possibly through an indirect mechanism [23]. Therefore, BCP may represent a promising treatment for several metabolic disorders.

The effects of BCP on adipose tissue have never been addressed before, and so far, all studies on the BCP effects on glucose metabolism have been reported only in vivo. In vitro studies are a fundamental step for initial screening of potential cellular targets and characterization of the cellular mechanisms of bioactive molecules. Moreover, in the aim of a dietary approach, natural plant extracts with a high and standardized content of BCP could have a more suitable impact with respect to plain/synthetic BCP. Therefore, the present study was designed to evaluate the chemical characterization of a black pepper extract with a high content of BCP (PipeNig^®^-FL) produced by Biosfered Srl (Italy), and to study its effects on lipid accumulation and glucose uptake in in vitro models. The efficacy of the product was evaluated in two different cellular models, 3T3-L1 and C2C12, by assessing triglycerides accumulation in adipocytes and both glucose uptake and GLUT4 translocation in skeletal muscle myotubes.

## 2. Materials and Methods

### 2.1. Reagents

PipeNig^®^-FL (batch number PNF01-1907001), a *Piper nigrum* L. (black pepper) liquid extract, was kindly provided by Biosfered Srl (Torino, Italy). Certificate of analysis, technical sheets and materials safety data sheet of PipeNig^®^-FL are available from Biosfered upon request. The method of extraction and production of PipeNig^®^-FL are covered by the company trade secrets. PipeNig^®^-FL contains BCP at a concentration of 3.5 M in rice oil. For experiments, a stock solution of 1 M in DMSO was obtained, then diluted in culture medium for cell treatments. Concentrations reported in this work refer to those of BCP in each dilution.

NucBlue™ Live ReadyProbes™ Reagent and 2-(7-Nitrobenz-2-oxa-1,3-diazol-4-yl)Amino)-2-Deoxyglucose) (2-NBDG) were obtained from Invitrogen (Carlsbad, CA, USA); CellTiter-Glo^®^ Luminescent Cell Viability and CellTiter 96^®^ AQueous One Solution Cell Proliferation Assays were from Promega (Madison, WI, USA); anti-GLUT4 primary antibody and anti-rabbit secondary antibody Alexa Fluor 568 were from ThermoFisher Scientific (Waltham, MA, USA). Human insulin was used for cell treatments. Unless otherwise specified, all chemicals were purchased from Sigma Aldrich (St. Louis, MO, USA).

### 2.2. Gas-Chromatographic Analyses of PipeNig^®^-FL

PipeNig^®^-FL was analyzed by gas-chromatography (mod. 6890N, Agilent Technologies, Santa Clara, CA, USA) coupled with mass spectrometry (mod. 5973A, Agilent Technologies) (GC–MS). Compounds were separated on a Zebron ZB-5MS (mod. 7HG-G010-11, Phenomenex, Torrance, CA, USA) capillary column (stationary phase: 95% polydimethyl siloxane—5% diphenyl, 30 m length, 250 µm internal diameter, 0.25 µm film thickness) with the following temperature program: 60 °C for 5 min followed by a temperature rise at a 3 °C min^−1^ rate to 270 °C (held for 5 min). Carrier gas was He with a constant flow of 1 mL min^−1^, transfer line temperature to MSD was 280 °C, ionization energy (EI) 70 eV, and full scan range 50–300 m/z. Separated compounds were identified by pure standard comparison, by comparison of their mass spectra with those of reference substances and by comparison with the NIST mass spectral search software v2.0 using the libraries NIST 98 library. Quantitative analyses were confirmed by gas chromatography coupled with flame ionization detector (GC–FID) (mod. 6890N, Agilent Technologies); analyses performed with the same column and GC conditions as above.

### 2.3. Cell Cultures

3T3-L1 preadipocytes (ATCC^®^ CL-173™; Lot No 70009858, ATCC, Manassas, VA, USA) were cultured in high-glucose (4.5 g/L) Dulbecco’s modified Eagle’s medium (DMEM) supplemented with 10% calf serum, 2 mM L-glutamine, 50 IU/mL penicillin, and 50 μg/mL streptomycin [24]. For experiments, 5 × 10^3^ cells/well were seeded in 96-black well clear bottom plates (Greiner Bio-One, Frickenhausen, Germany). Two days after reaching confluence (day 0), cells were exposed to the differentiation medium (MDI; which was DMEM containing 10% fetal bovine serum (FBS), 1 μg/mL insulin, 0.25 μM dexamethasone, 0.5 mM isobutylmethylxanthine). Two days later (day 2), MDI was replaced with maintenance medium (MM; which was DMEM 10% FBS, 1 μg/mL insulin). Fresh medium was provided every two days. Experiments ended after 9 days from the beginning of the differentiation (day 9). 

The mouse myoblast cell line C2C12 (ECACC 91031101, lot 17I044) was purchased from the European Collection of Authenticated Cell Cultures (ECACC, Salisbury, UK) and cultured in high-glucose DMEM supplemented with 10% FBS, 1% penicillin/streptomycin and 2 mM L-glutamine in a humidified atmosphere of 5% CO_2_ at 37 °C. Cultures were plated at a density of 2 × 10^3^ cells per cm^2^ on tissue plastic dishes (Becton Dickinson, Franklin Lakes, NJ, USA) and sub-cultured before reaching 70% confluence. For experiments, cells were seeded at a density respectively of 2 × 10^3^ cells/cm^2^ in 96-well plates or 10 × 10^3^ cell/cm^2^ on coverslips or glass bottom dishes (VWR Int., Radnor, PA, USA), to enhance adhesion. After cells reached confluence, differentiation was induced by changing the medium to DMEM supplemented with 2% horse serum (HS). Cells were allowed to differentiate for additional 5 to 7 days. The day before glucose uptake and GLUT4 translocation experiments, C2C12 cells were starved in DMEM glucose and serum free for 24 h.

### 2.4. Cell Viability

The viability of 3T3-L1 cells was evaluated at the end of the experiments (day 9) by CellTiter-Glo^®^ Luminescent Cell Viability Assay, based on the quantitation of ATP, which signals the presence of metabolically active cells. After AdipoRed™/NucBlue™ quantification (see belove), the dye mixture was removed from the cell cultures and CellTiter-Glo^®^ reagent, diluted 1:1 in phosphate-buffered saline (PBS), was added. Cells were incubated at room temperature in the dark for 10 min, then luminescence was detected and quantified with FilterMax F5™ Multi-Mode microplate reader (Molecular Devices, Sunnyvale, CA, USA). The values of luminescence are directly proportional to the number of viable cells. Data from three independent experiments were expressed as percentage referred to control condition; these values were then summarized to calculate mean ± standard error of the mean (SEM).

C2C12 cell viability was evaluated by the CellTiter 96^®^ AQueous One Solution Cell Proliferation Assay, using the tetrazolium compound [3-(4,5-dimethylthiazol-2-yl)-5-(3-carboxymethoxyphenyl)-2-(4-sulfophenyl)-2H-tetrazolium, inner salt (MTS), that is bioreduced by metabolically active cells into a colored formazan product soluble in tissue culture medium. C2C12 cells, grown and differentiated into 96-well plates, were treated in 50 µL DMEM + 2% HS with different concentrations of PipeNig^®^-FL for one hour; during the last 30 min, 10 µL of MTS were added to each well (six wells for each condition). Formazan product was measured with FilterMax F5 microplate reader at 450 nm; absorbance is directly proportional to the number of viable cells. Data from three independent experiments were expressed as percentage referred to control condition; these values were then summarized to calculate mean ± SEM.

### 2.5. Quantification of Adipocyte Lipid Accumulation and DNA Staining

The 3T3-L1 cells, grown in 96-black well clear bottom plates, were exposed to PipeNig^®^-FL from day 0 to day 9 (whole differentiation period treatment), at scalar dilutions ranging from 1 nM to 10 µM (maximum DMSO concentration: 0.1%). Control cells were treated with 0.1% DMSO. Experiments were repeated three times (four wells for each condition), using cells at different passage numbers (p3–p5). At the end of the experiments (day 9 after the induction of differentiation), lipid accumulation was quantified by using AdipoRed™ assay reagent (Lonza, Walkersville, MD, USA), while the DNA content was estimated by NucBlue™ staining. Briefly, medium was removed from 3T3-L1 cultures and cells were rinsed with PBS, subsequently replaced with a dye mixture containing AdipoRed™ and NucBlue™ assay reagents diluted in PBS (25 µL and 1 drop, respectively, per mL of PBS). After 40 min of incubation at room temperature in the dark, fluorescence was measured with Filtermax F5 microplate reader respectively with excitation at 485 nm and emission at 535 nm for AdipoRed™ and excitation at 360 nm and emission at 460 nm for NucBlue™ quantification. Data from three independent experiments were expressed as percentage referred to control condition; these values were then summarized to calculate mean ± SEM.

### 2.6. Glucose Uptake Measurements

C2C12 cells, plated and differentiated on glass bottom dishes, after 24 h without glucose and serum, were treated with a different concentration of PipeNig^®^-FL (1–10–100 nM), and simultaneously loaded with 100 µM of 2-NBDG in glucose and serum-free DMEM, for 30 min in the dark. Insulin (25 nM) was used as a positive control. After two washes in PBS, cells were observed in confocal microscopy. Fluorescence images at 488 nm were acquired using an Olympus Fluoview 200 laser scanning confocal system (Olympus America Inc., Melville, NY, USA) mounted on an inverted IX70 Olympus microscope, equipped with a 60X Uplan FI (NA 1.25) oil-immersion objective. Fluorescence variations were calculated with the definition and measurement of regions of interest (ROIs) using the ImageJ software (Rasband, W.S., U. S. National Institutes of Health, Bethesda, MA, USA; http://rsb.info.nih.gov/ij/, 1997–2008). Data from four independent experiments were evaluated as mean fluorescence/area and expressed as percentage referred to control condition; these values were then summarized to calculate mean ± SEM.

### 2.7. GLUT4 Translocation Analysis

C2C12 cells were grown and differentiated on glass coverslips. After 24 h without glucose and serum, cells were treated with insulin 25 nM or different concentration of PipeNig^®^-FL (1–10–100 nM) for 30 min in glucose and serum-free DMEM. Then cells were fixed for 40 min in 4% paraformaldehyde dissolved in 0.1 M phosphate buffer, pH 7.3. After three washes with PBS, cells were incubated for 20 min with 0.3% Triton and 1% bovine serum albumin in PBS and stained for 24 h at 4 °C with the primary polyclonal antibody anti-GLUT4, 1:100. Cover slides were washed twice with PBS and incubated for 1 h at room temperature with the secondary antibody, anti-rabbit Alexa Fluor 568, 1:1000. After two washes in PBS, coverslips were mounted on standard slides with DABCO and observed after 24 h under confocal microscope. GLUT4 staining measurements of both cell periphery and cell interior were performed with the ABSnake plugin of the ImageJ software [25]. Briefly, for each myotube the ABSnake plugin was employed to design a ROI band of 1.45 µm around the plasma membrane and the fluorescence intensities of both the band and the cellular inside were collected. Data from three independent experiments were expressed as peripheral/internal fluorescence and summarized to calculate mean ± SEM.

### 2.8. Statistical Analysis

Data are expressed as mean ± standard error of the mean (SEM); statistical analysis was performed using ANOVA (one-way analysis of variance) followed by Bonferroni’s multiple comparison test. Differences with *p* < 0.05 were considered statistically significant.

## 3. Results

### 3.1. Chemical Composition of PipeNig^®^-FL

**Figure 1:**
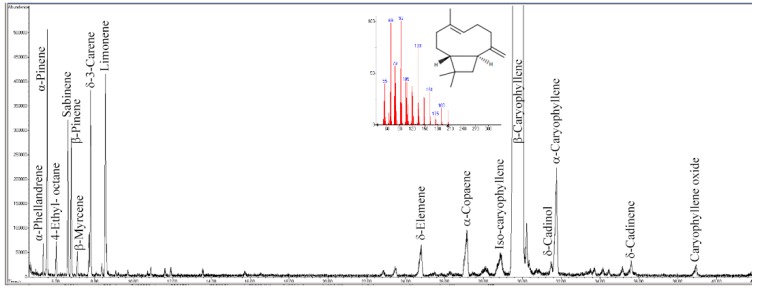
Gas chromatography–mass spectrometry (GC–MS) total ion current gas-chromatogram of PipeNig^®^-FL. The main compound *trans*-β-caryophyllene (BCP) is out of scale in order to evidence the other minor monoterpenes and sesquiterpenes that characterize the chemical composition of PipeNig^®^-FL. The inset shows the chemical formula and the mass spectrum of BCP. The y axis is the total ion current; the x axis represents time (in min).

The chemical composition of PipeNig^®^-FL was assessed by GC–MS and quantified by GC–FID, as specified in the Materials and Methods section. The extract is characterized by a high content and percentage of the sesquiterpene hydrocarbon BCP, followed by minor mono and sesquiterpenes as depicted in Figure 1 and listed in Table 1. In particular, the total standardized content of PipeNig^®^-FL was higher than 800 mg g^−1^ of product, in agreement with what is specified by the producer. Amounts ranging from 0.5 to 7.8 mg g^−1^ were represented by monoterpenes (with limonene being the most abundant), whereas, among sesquiterpenes, α-caryophyllene showed the highest amount. In terms of relative percentage, BCP reached a level of almost 88%, whereas total percentage of all other identified compounds was around 8% (Table 1).

Concentrations reported in this work for experiments with cell cultures refer to those of BCP contained in each dilution of PipeNig^®^-FL.

### 3.2. Effects of PipeNig^®^-FL on 3T3-L1 Adipocyte Cell Viability

**Figure 2:**
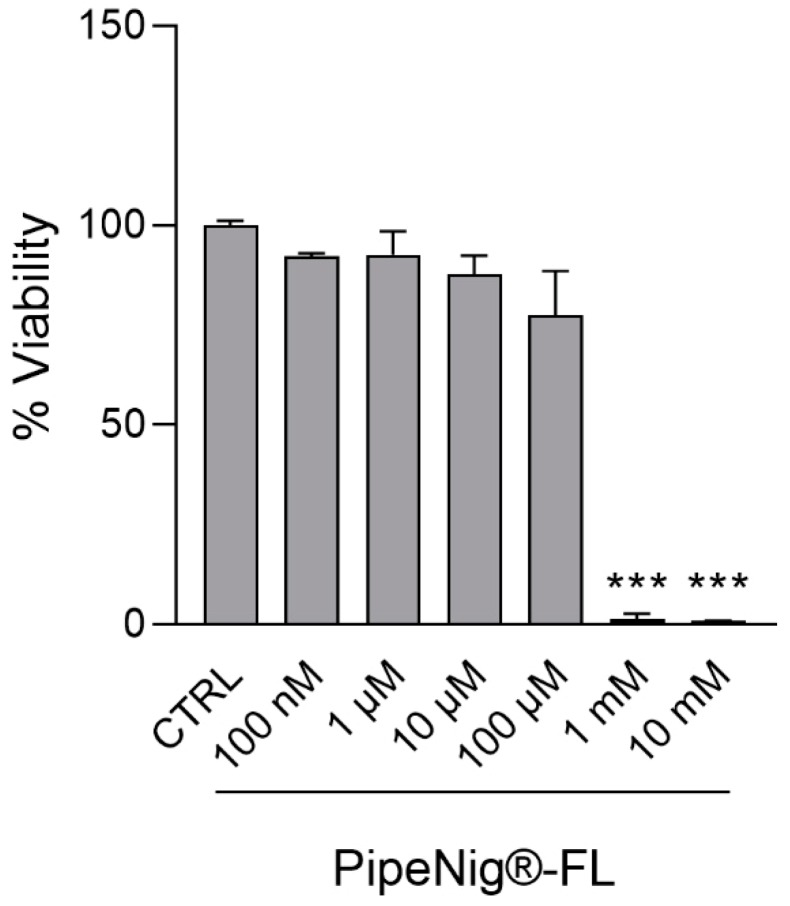
PipeNig^®^-FL affects 3T3-L1 cell viability only at high (millimolar) concentrations. 3T3-L1 cells were induced to differentiate into adipocytes for 9 days and treated with increasing concentrations of PipeNig^®^-FL for the entire differentiation period. The bar graph summarizes cell viability based on ATP content. Data in percentage referred to control condition are represented as the mean ± standard error of the mean (SEM) of three independent experiments. *** *p* < 0.001 vs. control.

The long-term viability of 3T3-L1 cells treated with a wide range of PipeNig^®^-FL concentrations (100 nM, 1 µM, 10 µM, 100 µM, 1 mM, 10 mM) was determined by the CellTiter-Glo^®^ viability assay, a method based on measurement of ATP content, whose amount is directly proportional to the number of metabolically active cells present in culture. As shown in Figure 2, after 9 days from the beginning of adipocyte differentiation (see Materials and Methods section), cell viability was affected only at very high PipeNig^®^-FL concentrations (1 mM and 10 mM), with only a minor, not statistically significant decrease at 100 μM. Based on these results, concentrations up to 10 μM were chosen for subsequent experiments. Mean values of luminescence signal from three independent experiments were as follow: CTRL (DMSO 0.1%): 100 ± 0.20, PipeNig^®^-FL 100 nM: 92.31 ± 0.13, PipeNig^®^-FL 1 µM: 92.65 ± 1.01, PipeNig^®^-FL 10 µM: 87.86 ± 0.83, PipeNig^®^-FL 100 µM: 77.55 ± 2.12, PipeNig^®^-FL 1 mM: 1.46 ± 1.32, PipeNig^®^-FL 10 mM: 0.93 ± 0.12.

### 3.3. PipeNig^®^-FL Reduces Intracellular Lipid Accumulation in 3T3-L1 Cells without Altering the Cell Number

**Figure 3:**
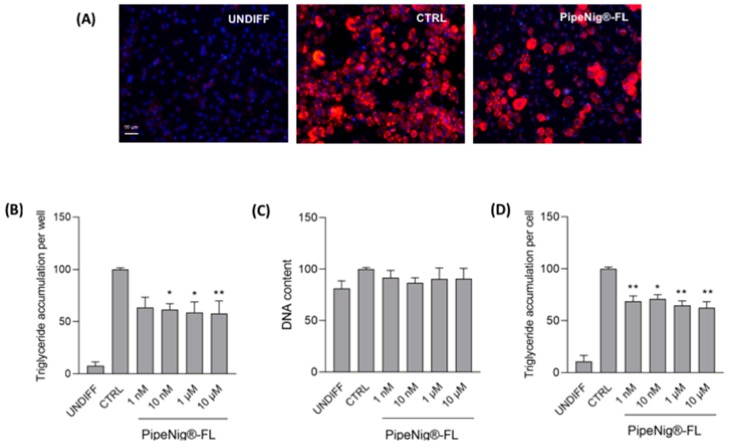
PipeNig^®^-FL reduces intracellular lipid accumulation in 3T3-L1 cells without altering the cell number. (**A**) Representative images of AdipoRed (red) and NucBlue (blue) staining of undifferentiated preadipocytes (UNDIFF), differentiated control adipocytes (CTRL) and 10 µM PipeNig^®^-FL-treated 3T3-L1 adipocytes after 9 days of differentiation. Scale bar 50 µm. (**B**) Bar graph summarizing AdipoRed staining experiments to assess lipid accumulation on undifferentiated cells, differentiated control and 3T3-L1 adipocytes treated with various concentrations of PipeNig^®^-FL for 9 days, showing triglyceride accumulation per well. (**C**) Bar graph summarizing NucBlue staining experiments to assess variations in the number of cells, showing DNA content per well. (**D**) Bar graph showing triglyceride accumulation per cell, calculated as the ratio of AdipoRed and NucBlue staining. Data in percentage referred to differentiated control condition are represented as the mean ± SEM of three independent experiments. * *p* < 0.05; ** *p* < 0.01 vs. control.

The potential antiadipogenic activity of PipeNig^®^-FL was assayed on the murine 3T3-L1 preadipocyte cell line, a commonly used cell model for adipose cell biology research [26]. Since antiadipogenic effects can be exerted by reducing both intracellular lipid accumulation and/or the number of adipocytes (either by decreasing cell proliferation or inducing cell death), we simultaneously assayed triglyceride accumulation (AdipoRed^TM^ assay) and cell number (NucBlue^TM^ staining, measuring DNA content). Confluent preadipocytes, cultured in 96-well plates, were induced to start adipogenic differentiation and were treated throughout the differentiation period (9 days) with a vehicle only (0.1% DMSO; differentiated control) or with 1 nM, 10 nM, 1 µM, 10 µM PipeNig^®^-FL. Higher PipeNig^®^-FL doses were not used, based on the cell viability data reported above. After 9 days from the beginning of adipocyte differentiation, AdipoRed^TM^/NucBlue^TM^ stainings were performed on 3T3-L1 adipocytes (Figure 3A). Trygliceride accumulation and DNA content were calculated as percentage change from differentiated DMSO-treated controls (Figure 3B,C). The DNA content was used to normalize total triglyceride values to obtain triglyceride content per unit DNA (as a proxy for triglycerides accumulation per cell).

As shown in (Figure 3B), triglyceride accumulation per well was reduced in PipeNig^®^-FL treated 3T3-L1 cells compared to differentiated control cells; in particular, statistically significant reductions were obtained after treatment with PipeNig^®^-FL 10 nM, 1 µM and 10 µM. Mean values of fluorescence signal from three independent experiments were as follow: undifferentiated cells: 7.85 ± 2.36, differentiated CTRL: 100 ± 0.20, PipeNig^®^-FL 1n M: 63.64 ± 2.37, PipeNig^®^-FL 10 nM: 61.72 ± 1.35, PipeNig^®^-FL 1 µM: 59.00 ± 2.42, PipeNig^®^-FL 10 µM: 57.91 ± 3.05.

DNA content was not significantly different in PipeNig^®^-FL treated cells and control cells, thus indicating that the decrease in lipid accumulation exerted by PipeNig^®^-FL is not due to a decrease in cell proliferation or to cytotoxic effects (Figure 3C). Mean values of fluorescence signal from three independent experiments were as follows: undifferentiated cells: 81.23 ± 1.36, differentiated control: 100 ± 0.20, PipeNig^®^-FL 1n M: 91.63 ± 1.39, PipeNig^®^-FL 10 nM: 86.76 ± 0.96, PipeNig^®^-FL 1 µM: 90.43 ± 2.03, PipeNig^®^-FL 10 µM: 90.72 ± 1.92.

On the other hand, a significant reduction in intracellular lipid content per cell was found at all concentrations (Figure 3D); mean values of fluorescence signal from three independent experiments were as follow: undifferentiated cells: 10.63 ± 3.62, differentiated CTRL: 100 ± 0.30, PipeNig^®^-FL 1 nM: 68.68 ± 1.12, PipeNig^®^-FL 10 nM: 71 ± 0.88, PipeNig^®^-FL 1 µM: 64.74 ± 1.01, PipeNig^®^-FL 10 µM: 62.57 ± 1.31.

### 3.4. PipeNig^®^-FL Does Not Affect Cell Viability on C2C12 Muscle Cell

**Figure 4:**
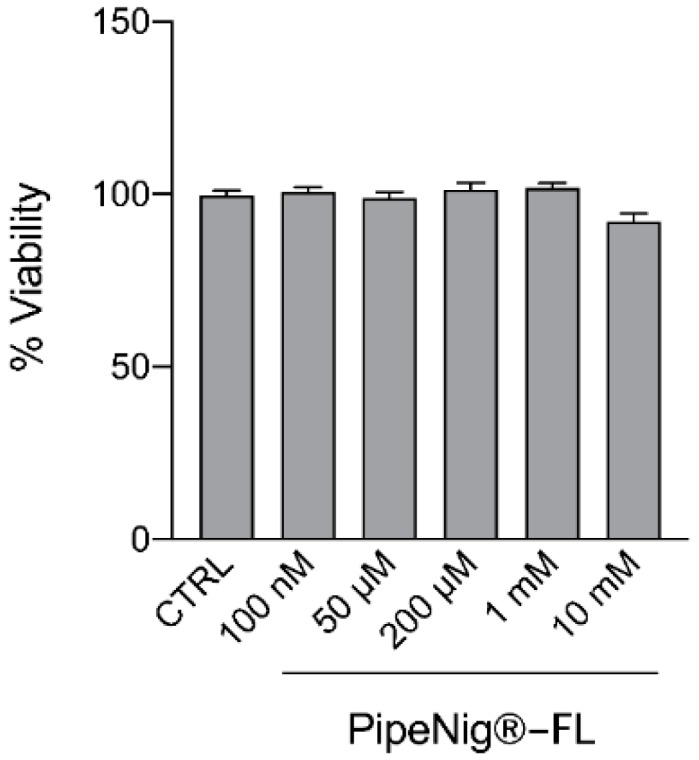
PipeNig^®^-FL does not have any effects on cell viability in C2C12 muscle cell. C2C12 cells were treated with increasing concentration of PipeNig^®^-FL for 1h. Data in percentage referred to control condition are represented as the mean ± SEM (*n* = 3).

In order to investigate the effect of PipeNig^®^-FL on C2C12 viability, differentiated cells were treated with increasing concentration of PipeNig^®^-FL (100 nM, 50 µM, 200 µM, 1 mM, 10 mM) for 1 h. The MTS assay was performed in the last 30 min of treatment. The time considered to evaluate the toxicity of PipeNig^®^-FL was related to experiments on glucose metabolism that were done in a short time of treatment. As shown in Figure 4, acute exposure (1 h) to PipeNig^®^-FL even at the highest doses did not affect significantly (*p* < 0.05) cell viability. Mean values of Abs at 450 nm from three independent experiments were as follows: CTRL: 99.61 ± 1.48, PipeNig^®^-FL 100 nM: 100.72 ± 1.32, PipeNig^®^-FL 50 µM: 98.90 ± 1.78, PipeNig^®^-FL 200 µM: 101.27 ± 2.04, PipeNig^®^-FL 1 mM: 101.76 ± 1.39, PipeNig^®^-FL 10 mM: 92.13 ± 2.34.

### 3.5. PipeNig^®^-FL Improves Glucose Uptake in C2C12 Myotubes

**Figure 5:**
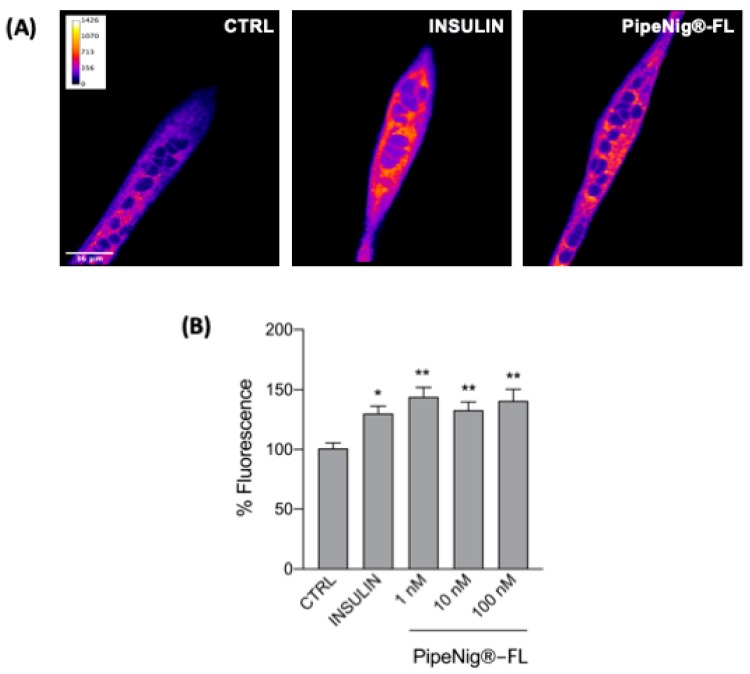
PipeNig^®^-FL stimulates glucose uptake. (**A**) Representative confocal images of C2C12 myotubes incubated with the fluorescent glucose analog 2-NBDG for 30 min. Images are presented in pseudocolor (LUT = fire) to better show the fluorescence intensity variations. Insulin (25 nm) was used as a positive control. Scale bar 36 µm. (**B**) Bar graph summarizing the experiments of fluorescent glucose uptake. Data in percentage referred to control condition are represented as the mean ± SEM (*n* = 4). * *p* < 0.05; ** *p* < 0.01 vs. control.

To verify the potential role of PipeNig^®^-FL on glucose uptake in skeletal muscle cells, we performed confocal microscopy analyses by using a fluorescent D-glucose analog, 2-NBDG. Cells were incubated simultaneously with either 100 µM 2-NBDG and different concentrations of PipeNig^®^-FL (1–10–100 nM) without insulin while insulin alone (25 nM) was used as positive control, for 30 min in the dark. Doses of PipeNig^®^-FL were chosen in the nM range as for insulin, since we have previously verified that stimulation with PipeNig^®^-FL 100 nM for 1 h did not produce cytotoxic effects. A significant increase of glucose uptake was observed in treated cells with respect to control cells, whereas no differences were observed between insulin and PipeNig^®^-FL treatments or among PipeNig^®^-FL concentrations (Figure 5). Values of mean fluorescence/area from four independent experiments were as follows: CTRL: 100.13 ± 4.69, n cells = 68; insulin: 130.10 ± 6.21, n cells = 89; PipeNig^®^-FL 1 nM: 144.04 ± 7.95, n cells = 45; PipeNig^®^-FL 10 nM: 133.00 ± 6.73, n cells = 75; PipeNig^®^-FL 100 nM: 140.72 ± 9.64, n cells = 56.

### 3.6. PipeNig^®^-FL Induces GLUT4 Translocation in C2C12 Cells

**Figure 6:**
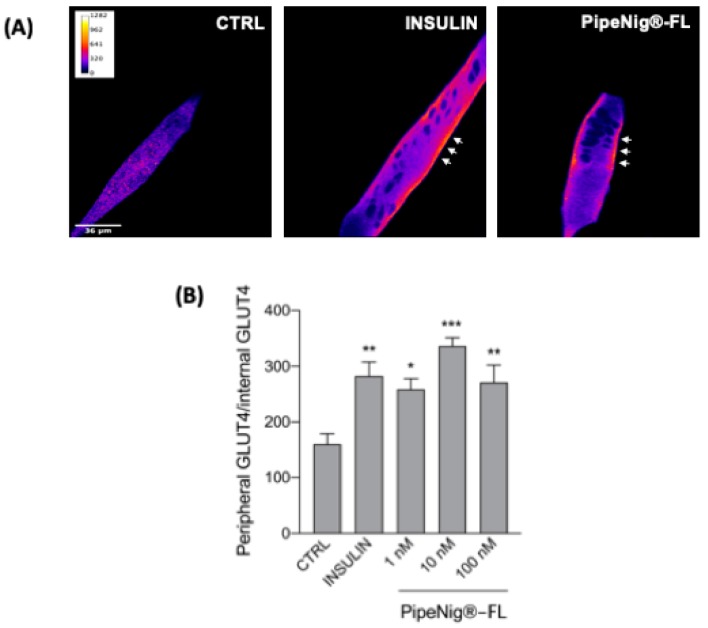
PipeNig^®^-FL induces GLUT4 translocation to the plasma membrane. (**A**) Confocal images of a representative experiment of GLUT4 immunofluorescence staining. After PipeNig^®^-FL stimulation (1–10–100 nM) the fluorescent signal is clearly localized to the peripheral plasmalemma, thus suggesting the GLUT4 translocation. Images are presented in pseudocolor (LUT = fire) to better show the fluorescence intensity variations. Insulin (25 nM) was used as a positive control. Scale bar 36 µm. (**B**) Bar graph representing the ratio peripheral vs. internal GLUT4 fluorescence intensity. Data are represented as the mean ± SEM of three independent experiments. * *p* < 0.05; ** *p* < 0.01; *** *p* < 0,001 vs. control.

To confirm the involvement of PipeNig^®^-FL on glucose metabolism, we carried out immunofluorescence experiments using GLUT4 antibody, followed by a detailed image analysis of peripheral vs. internal fluorescence staining. Cells were treated with insulin (25 nM) or different concentrations of PipeNig^®^-FL (1–10–100 nM) without insulin, for 30 min. An evident translocation of the glucose transporter from the cytosol to the plasma membrane was observed in treated cells with respect to control cells, whereas there were no significant differences in staining among treatments (Figure 6). Values of peripheral vs. internal GLUT4 staining from three independent experiments were as follows: CTRL: 160.36 ± 18.21, n cells = 19; insulin: 282.73 ± 25.00, n cells = 21; PipeNig^®^-FL 1 nM: 259.26 ± 18.60, n cells = 20; PipeNig^®^-FL 10 nM: 336.40 ± 14.96, n cells = 18; PipeNig^®^-FL 100 nM: 270.96 ± 31.36, n cells = 16.

## 4. Discussion

This study focuses on two goals: 1. the chemical characterization of a black pepper extract with a high content of BCP (PipeNig^®^-FL). 2. the in vitro investigation of the biological activities of PipeNig^®^-FL in adipocytes and skeletal myotubes. The present results highlight the high performance of the extract regarding its BCP content (>80%) and underline its beneficial metabolic properties, in terms of reduced lipid accumulation in adipocytes and improved glucose uptake activity in myotubes.

The attractiveness of BCP, a sesquiterpene produced by a consistent number of plant species, arises from its pharmacological feature as a CB2 receptor full agonist, which makes BCP the only phytoendocannabinoid found beyond the *Cannabis* genus to date [27], with the advantage of lacking any psychotropic effect. In addition to CB2 receptors, BCP also activates peroxisome proliferator-activated receptor α and γ (PPARα-γ) [22,23], making it suitable to interfere with several metabolic pathways and pathological conditions, including apoptotic, inflammatory, cholesterolemic and behavioral disorders. In line with this, BCP has been demonstrated to possess anticancerogenic, neuroprotective, cardioprotective, hepatoprotective, gastroprotective, nephroprotective, antiinflammatory and immunomodulatary properties [27,28]. In particular, several studies recently pointed out potential counteractive functions of BCP against metabolic syndrome [16,17,18,19,29]. Grounding on these premises, the use of a plant-derived extract with a high and standardized BCP content represents a high-impact goal in the cross-sectional fields of plant food-nutrition-human health. In this perspective, the objective of our studies was to evaluate the properties of PipeNig^®^-FL to reduce lipid accumulation and induce glucose uptake.

The chemical analysis of PipeNig^®^-FL reveals a high content of BCP, in line with what is declared by the producer. The general GC profile is in line with the chemical composition of a typical black pepper oil [30,31] and confirms the presence of minor monoterpenes and sesquiterpenes as well as the complete absence of piperine.

We verified the suitability of PipeNig^®^-FL for in vitro cellular studies, by performing viability tests on 3T3-L1 preadipocytes and C2C12 myotubes. As shown in Figure 2, long-term 3T3-L1 treatment with PipeNig^®^-FL (9 days) did not cause any cell damage in the physiologically active range (100 nM–100 µM), only affecting cell viability at much higher concentrations (1–10 mM), while C2C12 viability (Figure 4) was unaffected by acute exposure to PipeNig^®^-FL (1 h, 100 nM–10 mM). Moreover, a previous study in mice reported the absence of toxicity of both acute (300 and 2000 mg/kg) and repeated doses of BCP [20], and BCP has been approved by United States Food and Drug Administration and European agencies as food additive, taste enhancer and flavoring agent [7].

We further tested the properties of PipeNig^®^-FL as a lipid accumulation-inhibitor during the 3T3-L1 cell differentiation process. The development of obesity is characterized by an increase in the number of fat cells (hyperplasia) and their lipid content (hypertrophy), as a result of cell proliferation and differentiation. In our experiments PipeNig^®^-FL led to a significant decrease in the lipid content per cell, without affecting cell proliferation, thus suggesting a role in reducing adipocyte-hypertrophic response typically present in the energy overload conditions that characterizes metabolic syndrome [32].

Previous in vitro studies on bone marrow mesenchymal stem cells showed enhanced osteoblast differentiation and reduced adipogenesis induced by synthetic BCP [33]. Moreover, recent in vivo studies in rats exposed to fat enriched diet highlighted hypocholesterolemic and protective effects of BCP [16,23], proving the involvement of several mechanisms, as the inhibition of endogenous hepatic cholesterol synthesis [16], the stimulation of the activity of antioxidant enzymes [16] and the engagement of both CB2 and PPARγ receptors [16]. In this scenario, our results of reduced adipogenesis induced by PipeNig^®^-FL in 3T3-L1 pre-adipocytes strengthen the message from data obtained with synthetic BCP in bone marrow cells, and support the effectiveness of the extract respect to its high BCP content.

In addition to its anti-obesogenic properties, our further experiments on skeletal myotubes highlighted PipeNig^®^-FL capability to be a glucose uptake inducer: as shown in Figure 5, PipeNig^®^-FL was as efficient as insulin in stimulating cellular glucose uptake. In 2014 Basha and Sankaranarayanan [18] reported an insulinomimetic effect of BCP (200 mg/kg) in streptozotocin-induced diabetic rats. In particular they showed a significant decrease in blood glucose levels and a significant increase in the activity of hexokinase, pyruvate kinase and glucose-6-phosphate dehydrogenase in liver, kidney and skeletal muscle [18]. Moreover, other studies in similar animal models [19] and in isolated pancreatic beta cells [34] reported antidiabetic properties of BCP through an enhanced insulin release. To gain better understanding of the mechanisms involved in glucose uptake, we performed immunofluorescence GLUT4 staining in insulin and PipeNig^®^-FL-treated myotubes, showing a significant plasma membrane GLUT4 translocation in both conditions (Figure 6) respect to control. Thus the attractive novelty of these results is to show, for the first time, a direct acute effect of a BCP-enriched extract in promoting glucose uptake in skeletal myotubes, likely through an improvement of GLUT4 trafficking toward the plasma membrane. Translocation of GLUT4 storage vesicles to the plasma membrane, mainly in skeletal muscle and adipose tissue, is directly correlated with the ability to lower elevated blood glucose. Moreover, GLUT4 levels are significantly decreased in the skeletal muscle of type 2 diabetic patients and in insulin resistant patients [35]. The development of therapeutic compounds able to induce GLUT4 expression/translocation can thus improve insulin sensitivity and reduce insulin resistance. Several plant-derived bioactive molecules have been listed as stimulators of GLUT4 translocation and/or expression, among these resveratrol, chlorogenic acid, daidzein (an isoflavone found in soybeans), curcumin and astaxanthin, affecting different key-points in the intracellular cascade involved in vesicle trafficking [36]. As a major component of PipeNig^®^-FL, BCP could now be included in this list.

## 5. Conclusions

The main limitation of our study resides in the experimental models, since we tested the effects of PipeNig^®^-FL on cell lines of preadipocytes and skeletal myotubes. On the other hand, in vitro approaches account for important advantages, as results reflect direct effects of PipeNig^®^-FL on specific cellular processes, avoiding complex multi-organ interactions typical of in vivo models. At present, we do not provide a detailed analysis on the complex molecular/functional signatures of reduced lipidogenesis activated by PipeNig^®^-FL in 3T3-L1 cells. Future experiments should be directed to gain further information on the molecular mechanisms initiated by PipeNig^®^-FL and to confirm its properties as anti-lipidogenic and glucose uptake inducer in animal models.

In conclusion, giving its high content in BCP, PipeNig^®^-FL could represent a promising novel bioactive complex deserving both molecular investigations and in vivo studies in order to support its role as a beneficial metabolic modulator.

## Figures and Tables

**Table 1:** Chemical composition of PipeNig^®^-FL by gas chromatography coupled to mass spectrometry. Content is calculated based on gas chromatography with flame-ionization detection (GC–FID) analysis. R.T., retention time.

Compound	R.T.	Content (mg g^−1^)	Relative %
α-Phellandrene	5.35	0.47	0.11
α-Pinene	5.55	4.06	0.95
4-ethyl-octane	6.02	0.64	0.15
Sabinene	6.62	2.93	0.69
β-Pinene	6.79	2.76	0.65
β-Myrcene	7.11	1.10	0.12
δ-3-Carene	7.79	3.85	0.90
Limonene	8.55	7.84	1.10
δ-Elemene	24.79	2.21	0.52
α-Copaene	27.15	3.37	0.75
Isocaryophyllene	28.86	1.18	0.28
β-Caryophyllene	29.94	814.44	87.61
δ-Cadinol	31.49	0.70	0.16
α-Caryophyllene	31.75	6.13	1.43
δ-Cadinene	35.61	0.74	0.17
Caryophyllene oxide	38.94	0.79	0.18
